# Evaluation of IL-28B Polymorphisms and Serum IP-10 in Hepatitis C Infected Chimpanzees

**DOI:** 10.1371/journal.pone.0046645

**Published:** 2012-10-30

**Authors:** Babs E. Verstrepen, Natasja G. de Groot, Zwier M. A. Groothuismink, Ernst J. Verschoor, Rik A. de Groen, Willy M. Bogers, Harry L. A. Janssen, Petra Mooij, Ronald E. Bontrop, Gerrit Koopman, Andre Boonstra

**Affiliations:** 1 Department of Virology, Biomedical Primate Research Centre, Rijswijk, The Netherlands; 2 Department of Comparative Genetics and Refinement, Biomedical Primate Research Centre, Rijswijk, The Netherlands; 3 Department of Gastroenterology and Hepatology, Erasmus MC University Hospital, Rotterdam, The Netherlands; University of Sydney, Australia

## Abstract

In humans, clearance of hepatitis C virus (HCV) infection is associated with genetic variation near the IL-28B gene and the induction of interferon-stimulated genes, like IP-10. Also in chimpanzees spontaneous clearance of HCV is observed. To study whether similar correlations exist in these animals, a direct comparison of IP-10 and IL-28B polymorphism between chimpanzees and patients was performed. All chimpanzees studied were monomorphic for the human IL-28B SNPs which are associated with spontaneous and treatment induced HCV clearance in humans. As a result, these particular SNPs cannot be used for clinical association studies in chimpanzees. Although these human SNPs were absent in chimpanzees, gene variation in this region was present however, no correlation was observed between different SNP-genotypes and HCV outcome. Strikingly, IP-10 levels in chimpanzees correlated with HCV-RNA load and γGT, while such correlations were not observed in humans. The correlation between IP-10, γGT and virus load in chimpanzees was not found in patients and may be due to the lack of lifestyle-related confounding factors in chimpanzees. Direct comparison of IP-10 and IL-28B polymorphism between chimpanzees and patients in relation to HCV infection, illustrates that the IFN-pathways are important during HCV infection in both species. The Genbank EMBL accession numbers assigned to chimpanzees specific sequences near the IL-28B gene are HE599784 and HE599785.

## Background

Worldwide, an estimated 170 million people are chronically infected with the hepatitis C virus (HCV) [1] and are therefore at risk to develop liver diseases, like cirrhosis and hepatocellular carcinoma. Upon infection with HCV, only a minority of individuals can clear the virus spontaneously, while the majority of patients become chronically infected [2], [3]. In recent years, an important role for host factors has been documented in determining the progression towards chronicity of HCV as well as prediction of therapy-induced clearance of HCV [4]–[10]. Genetic variation on chromosome 19, within an intergenic region upstream of the IL-28B gene, encoding for the IFN lambda 3 protein (IFNλ3) correlates with both spontaneous as well as treatment-induced clearance [6], [8]. Furthermore, low plasma levels of interferon-gamma-inducible protein 10 (IP-10) prior to therapy predict treatment-induced clearance of HCV infection [7], [11]–[13] but whether these two factors interrelate is currently under intense debate. However, this is highly relevant since both parameters directly interfere with antiviral IFN-pathways.

Chimpanzees are not only the closest living evolutionary relatives of humans but also the only validated animal model to study HCV infection. Similar to humans, HCV in chimpanzees can either lead to a self-limiting infection or to viral persistence, and also in the chimpanzee, antigen specific cellular immune responses are believed to be important [14]–[16]. In patients, low pre-treatment IP-10 levels in serum and strong upregulation of IFN-stimulated genes (ISGs) during treatment in the liver were shown to correlate with successful IFNα based therapy [7], [9], [11], [12], [17]. However, in contrast to humans, standard IFNα-based therapy failed to reduce serum HCV RNA levels in chronically infected chimpanzees [18], and therefore mechanisms leading to treatment-induced sustained virological response (SVR) cannot be studied in these animals. The lack of clinical response to IFNα in chimpanzees was suggested to be similar to that in patients nonresponsive to therapy: a maximum induction of ISGs by HCV itself, rather than by treatment with exogenous IFNα [18].

Interestingly, also a weak association between the preferred IL-28B genotypes and low serum IP-10 baseline levels in patients chronically infected with HCV was recently reported [11], [19]. This suggests an interaction between IP-10 and IFNλ during the course of HCV infection in humans [12]. However, at present, a clear understanding of the mechanisms and their potential role in viral clearance is lacking. In this study, we aimed to evaluate and compare IP-10 and polymorphism near the IL-28B gene in chimpanzees and humans in relation to HCV infection to obtain better insight in the potential association between the two host factors in HCV infection.

We show that, however chimpanzees do not possess the same human SNPs near the IL-28B gene, allelic variation is present in this region in chimpanzees. No association was observed between IL-28B polymorphism and the course of HCV infection in chimpanzees. Furthermore, we report a positive correlation between peripheral levels of IP-10 and HCV RNA as well as γGT levels in chimpanzees, but not in patients. These findings demonstrate differences between HCV-infected chimpanzees and patients, which may impact pathophysiologic processes in the liver.

## Results

### No evidence found for the human SNPs associated with HCV clearance in chimpanzees

In humans, spontaneous as well as treatment-induced clearance was found to be associated with a series of SNPs upstream of the IL-28B gene [4]–[6], [8], [10]. To assess whether clearance of HCV in chimpanzees is associated with the same SNPs, genotype analysis of chimpanzee DNA was performed for rs8099917 and rs12979860.

The SNP rs8099917 consistently showed thymidine at this position on both chromosomes, indicating that all animals tested were homozygous rs8099917-TT carriers. In humans rs8099917-T is the preferred allele associated with HCV clearance. In addition, the chimpanzees were also genotyped for the rs12979860 SNP, and again no evidence was found for variance at this position. All animals tested had homozygous rs12979860-TT genotypes. In humans, the rs12979860-T allele is the so-called risk-allele associated with an increased risk for viral persistence. Thus, both IL-28B associated human SNPs are not present in chimpanzees.

### Chimpanzees possess a unique sequence near the IL-28B gene

Since rs8099917 and rs12979860 genotyping was performed by sequencing, the neighboring nucleotide sequences are also known, allowing us to compare this region with documented human genome sequences (National Institutes of Health, Bethesda, USA). Two different haplotypes were observed near rs12979860, HE599784 and HE599785. Notable was the strong uneven distribution: 62 out of 63 animals were heterozygous, carrying both HE599784 and HE599785. Only one animal was found to be homozygous for HE599785. These data suggest that an as yet unknown balancing selection may be operative on this region.

### No detectable IFNλ in serum from chimpanzees

In humans carrying the rs12979860-TT genotype, significant lower serum levels of IFNλ were documented in chronically infected patients as compared to patients with resolved infections [19], [20]. To investigate whether this difference could also be observed in chimpanzees, ELISA was performed on sera from chronically infected animals and animals that cleared the infection. Using commercially available immunoassays designed to detect human IL-28A/B and IL-29, we were unable to detect IFNλ in sera from any of the chimpanzees tested, neither in animals chronically infected with HCV nor animals that spontaneously cleared the infection. In the same assays, IL-28A/B and IL-29 could be detected in sera from patients with chronic HCV infection (data not shown; detection limit of the assays: 62.5 pg/ml and 8 pg/ml, respectively).

### IP-10 levels in serum correlate with viral load in HCV infected chimpanzees, but not in humans

**Figure 1:**
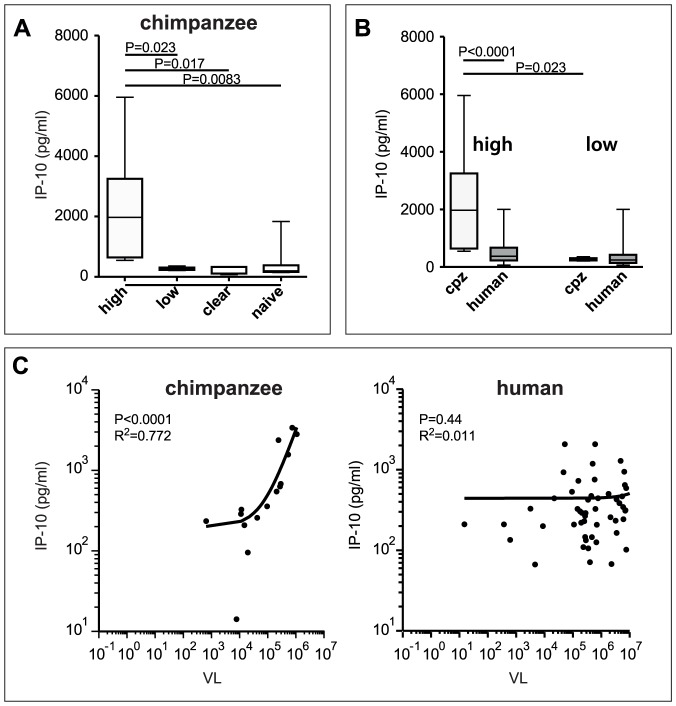
Correlation between HCV viremia and IP-10 in serum in chimpanzees. Box-whisker plots indicate the interquartile range and the median (horizontal line) of IP-10 concentrations in serum from animals of the different groups; “high HCV load” where the virus load of individual animal is higher as compared to median value of 200,000 IU/ml; “low HCV load” where the virus load of individual animal is lower as compared to the median value of 200,000 IU/ml; HCV resolvers and naïve, non-exposed (A). Box-whisker plots indicate the interquartile range and the median (horizontal line) of IP-10 concentrations in serum of humans and chimpanzees with high HCV load (>median virus load) and low HCV load (<median virus load) (B) and IP-10 concentrations plotted against HCV-RNA load in serum from chimpanzees and humans (C). A significant correlation between the two parameters is defined as r^2^>0.85 and p<0.05 where “r^2^” is a measure for correlation and “p” is a measure for the quality if this correlation.

To investigate whether the IP-10 levels in serum were associated with viral clearance, we compared IP-10 levels of chronically infected animals with a high HCV load (>200,000 IU/ml), low HCV load (<200,000 IU/ml), chimpanzees that had resolved HCV infection early after experimental exposure, and a group of HCV-naïve, non-exposed chimpanzees. As shown in Figure 1(a), the IP-10 levels in sera of animals with a high viral load were significantly higher relative to animals in the other groups. The serum IP-10 levels of animals with low viremia, animals that resolved the infection and HCV-naïve animals were relatively low and comparable between these groups.

Earlier studies in chronically infected patients demonstrated an association between viral load and IP-10 levels in serum [21], [22]. Therefore, we evaluated whether this correlation also existed in chimpanzees. As shown in Figure 1(b), at a low viral load, the IP-10 levels were similar in humans and chimpanzees. In contrast, serum IP-10 levels were higher in chimpanzees with a high viral load relative to human patients with similar high viral loads. A strong correlation between serum IP-10 concentration and viral load was observed in chronically infected chimpanzees. In contrast to chimpanzees, neither a correlation was observed in the overall group of patients chronically infected with HCV (Figure 1(c)), nor when this group was divided into subgroups according to their rs12979860 genotype (data not shown).

### Serum IP-10 concentrations correlate with γGT in HCV infected chimpanzees, but not in humans

**Figure 2:**
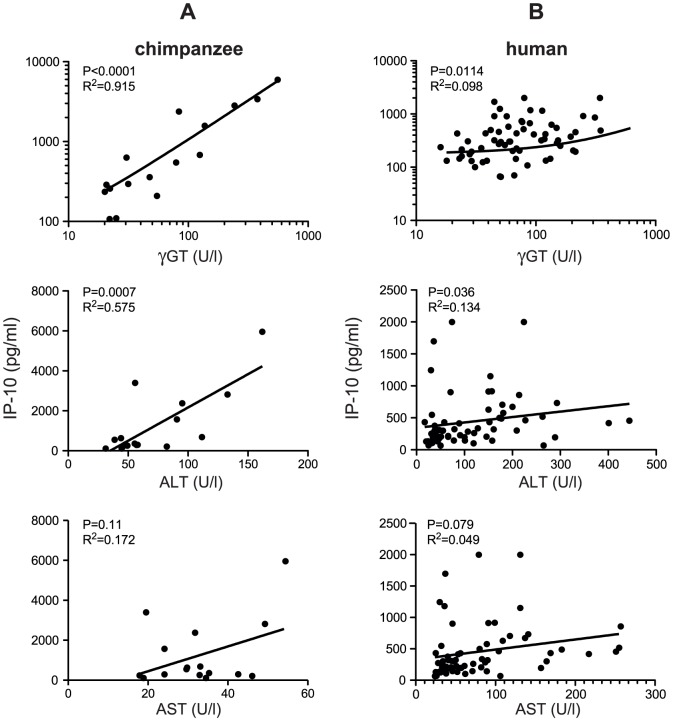
Relation between IP-10 levels and aminotransferases in serum. Relation between IP-10 and liver enzymes γGT, ALT and AST from HCV-infected individual chimpanzees (A) and HCV-infected patients (B) A significant correlation between the two parameters is defined as r^2^>0.85 and p<0.05 where “r^2^” is a measure for correlation and “p” is a measure for the quality if this correlation.

**Figure 3:**
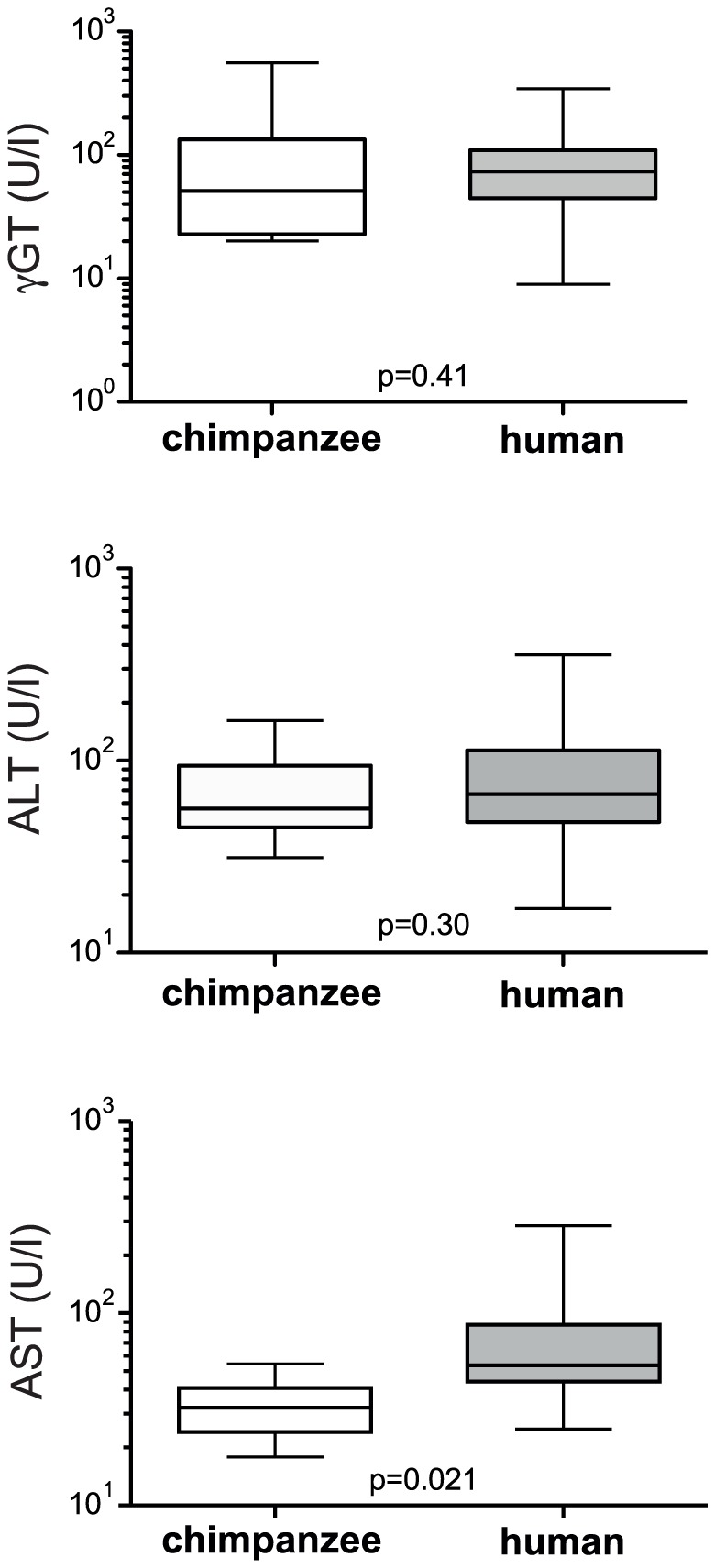
γGT, ALT and AST in humans and chimpanzees chronically infected with HCV. Interquartile range and the median (horizontal line) of γGT, ALT and AST in U/ml in chimpanzees and patients chronically infected with HCV.

In patients, the concentration of the serum aminotransferase ALT is used as indicator of active damaging processes in the liver during chronic HCV infection. However, in chimpanzees, γGT tends to be more increased rather than ALT. We therefore assessed whether IP-10 levels in blood correlated with the liver enzymes γGT, ALT and AST in chronically HCV infected chimpanzees and patients. As shown in Figure 2(a), in chimpanzees the serum levels of IP-10 strongly correlated with γGT (r^2^ = 0.91 and p<0.0001), while no correlation was observed between IP-10 and either ALT (r^2^ = 0.58; p = 0.0007) or AST (r^2^ = 0.17; p = 0.11). In contrast, in HCV infected patients, no correlations were observed between serum IP-10 and the aminotransferases (Figure 2(b)). Direct comparison of the levels of ALT and AST in serum of patients and chimpanzees demonstrated that only moderate ALT values were observed in chimpanzees, whereas 4 out of 58 patients exceeded an ALT value of 200 U/ml which is more then 4 times the maximum reference value for healthy humans and chimpanzees (Figure 3). Similarly, also AST levels were only moderately increased in chimpanzees, whereas 11 patients exceeded the maximum reference value by threefold. In contrast, serum γGT levels were found to be substantially elevated in some individuals from both species: in 3 out of 16 chimpanzees and 7 out of 60 patients, the γGT levels exceeded 200 U/ml, which is more then 5 times its reference value in healthy humans and chimpanzees.

## Discussion

In humans, HCV clearance is associated with specific IL-28B gene polymorphisms as well as the levels of specific ISG, such as IP-10 [4], [5], [7], [8], [10], [13]. It has been suggested that these pathways are associated with one another in humans [11], [19] although the interrelating mechanism is still unknown. The work presented here was designed to assess whether a potential correlation between IFNλ and polymorphism near the IL-28B gene can be studied in chimpanzees.

In humans, genetic variation near the IL-28B gene is associated with spontaneous as well as treatment-induced clearance of HCV [4], [5], [8], [10]. In chimpanzees, we found no evidence for these human SNPs and therefore it is unlikely that these specific SNPs play a role during HCV infection in chimpanzees. Instead, all animals tested were found to be homozygous carriers for rs12979860-TT and rs8099917-TT, which is in line with recently reported data on chimpanzees from another primate center [23]. A potential explanation may be that HCV is regarded as a human disease as no documentation is available on wild chimpanzees infected with HCV. In that respect different evolutionary selective pressure may have caused differences in innate responses between both species.

Since the documented SNPs, rs12979860 and rs8099917 are located in an intergenic region rather than within gene-encoding regions of the DNA, there may be a link between the region where the SNPs are located and another, yet unspecified gene. Given the fact that humans and chimpanzees share a common ancestor, the chimpanzee sequence likely represents the ancestral genotypes. This is in line with the finding that Central and Western African human populations carry the rs12979860-TT genotype at high frequency [10].

Even though humans and chimpanzees show 98.8% identity at the DNA level [24], many SNPs identified in humans are not necessarily present in chimpanzees. Although no evidence was found in chimpanzees for the two SNPs with documented relevance during HCV infection in humans, additional polymorphism near the IL-28B gene was detected. In the animals tested, a remarkable level of heterozygous carriers was observed, which may suggest a balancing selection being operative on this region. Our findings cannot be explained by the breeding strategy as concluded on the basis of variation in the mtDNA [25], and because it was also observed in animals from outside the BPRC breeding colony. The underlying mechanism responsible for this heterogeneity to assure a genetically diverse population is unknown.

In serum from chimpanzees no IFNλ was detected, and therefore no conclusion could be drawn from its effect on the outcome of HCV infection in chimpanzees. IFNλ has been measured in chimpanzees before using human detection reagents [23]. Given the rapid normalization of IFNλ levels in chimpanzees shortly after infection, it is expected that IFNλ is too low to detect during the chronic phase of infection. Lower IFNλ levels in chimpanzees could not be explained by a different copy number of the IFNλ-encoding genes as both in humans as well as chimpanzees only one copy of each of the IL-29/IL28A and IL-28B genes was observed (human and chimpanzee database supported by National Institutes of Health, Bethesda, USA).

In patients chronically infected with HCV, low baseline IP-10 levels are predictive for successful treatment-induced clearance [7]. Our data show that IP-10 levels are higher in chronically infected chimpanzees relative to patients. This finding is in line with the suggestion that HCV infection causes stronger upregulation of ISGs in chimpanzees as compared to humans [18]. Furthermore, based on data from a limited number of animals with high HCV-RNA levels, it was suggested that the failure of chimpanzees to respond successfully to IFN-based antiviral treatment was due to high baseline activation of the IFN-system. Based on our data it is to be expected that peripheral IP-10 levels from the animals in the Lanford-study are high. Given the documented correlation between high IP-10 and limited treatment success in humans, it is tempting to speculate that chimpanzees with a high HCV load and subsequently high baseline IP-10 levels in serum are indeed equivalent to human non-responders to IFN-based therapy. However, this implies that animals with lower IP-10 may respond to IFN-based therapy.

As humans and chimpanzees do not to show the same variation near the IL-28B gene, we were not able to confirm the earlier documented association between IL-28B variation and γGT levels in humans [17]. We did however find a correlation between γGT, IP-10 and virus load. This may imply, interrelating mechanisms play a role in both humans and chimpanzees, but that species-specific factors may contribute to biochemical differences between both species.

### Conclusion

Our data show that although chimpanzees do not possess the SNP near the IL-28B gene that are associated with HCV outcome in humans, chimpanzees do show genetic variation in this region. Furthermore, we found a positive correlation between IP-10 and viral load as well as γGT in chimpanzees, which was not found in patients. This difference may reflect the heterogeneous characteristics of HCV induced reactions in the liver of both species. The correlation between IP-10, virus load and γGT may reflect the lack of confounding factors in chimpanzees, since heavy alcohol intake, diabetes and obesity are known to influence the progression of HCV infection in humans, but not in chimpanzees.

## Methods

### Study population

To study the potential roles of IP-10 and polymorphisms during HCV infection in chimpanzees, we examined these parameters using the unique repository of chimpanzee DNA and serum samples of the Biomedical Primate Research Centre (BPRC). This database contains surplus material from blood samples collected routine health check purposes by both the BPRC as well as third party owners of chimpanzees. The work described here, was designed around the availability of the samples thus, no samples were collected from chimpanzees for the purpose of this work. Table 1 summarizes the relevant characteristics of the chimpanzee cohort included in this study. The animals were divided into different groups based on their HCV status: 1) “high viral load”: animals who were experimentally infected with HCV and virus load consistently higher than 200,000 IU/ml, where 200,000 is the median virus load of the animals in the study; 2) “low viral load”: animals who were experimentally infected with HCV and viral load consistently lower than 200,000 IU/ml; 3) “cleared”: animals who were experimentally infected with HCV and resolved infection 4) “naive“: non–infected animals that serve as healthy controls. In addition, DNA samples of 44 animals were screened for the presence of genetic variation near the IL28B gene. In total 63 chimpanzees were included in the analysis of the IL-28B region of which 11 animals were not part of the BPRC breeding.

**Table 1:** Characteristics of chimpanzee population.

	High viral load	Low viral load	Cleared HCV	Naive animals
**n**	8	6	6	10
**genotype**	1	1	1	
**years p.i.**	4 (4–21)	3 (2–4)	4.5 (3–10)	
**HCV RNA (IU/ml)**	505,000 (207,000–1,080,000)	31,908 (649–95,500)	0	0
**IP-10 (pg/ml)**	2247 (547–5956)	274 (209–359)	143 (71–131)	414 (146–1834)
**ALT (U/ml)**	91 (39–162)	58 (46–82)		
**AST (U/ml)**	34 (20–54)	33 (18–46)		
**γGT (U/ml)**	204 (30–558)	33 (20–55)		

Characteristics of the chimpanzee population studied, the HCV-genotype, the time since HCV exposure, HCV-RNA load, serum IP-10levels, and the liver enzymes levels ALT, AST and γGT. The average values and the range are shown.

Blood samples were obtained from patients with chronic HCV infection visiting the outpatient clinic of the Erasmus Medical Center (see Table 2). Patients were infected with either HCV genotype 1 (n = 37), genotype 2 (n = 3), genotype 3 (n = 13), genotype 4 (n = 4) or genotype 6 (n = 1). Patients co-infected with human immunodeficiency virus, hepatitis A virus, hepatitis B virus or hepatitis D virus were excluded from the study. The protocol was approved by the Medical Ethics Committee of the Erasmus Medical Center and all patients gave their written informed consent. In line with the chimpanzee cohort, the patients were divided into different groups based on their HCV status: 1) “high virus load”: virus load consistently higher than 500,000 IU/ml, where 500,000 is the median virus load of all patients in this study; 2) “low virus load”: patients with virus load consistently lower than 500,000 IU/ml.

**Table 2:** Characteristics of the patient cohort chronically infected with HCV.

	High viral load	Low viral load
**n**	30	27
**genotype**	1/2/3/6	1/3/4
**HCV RNA (IU/ml)**	7,827,576 (585,185– 42,100,000)	255,115 (15– 2,114,180)
**IP-10 (pg/ml)**	540 (67–2000)	281 (66–902)
**ALT (U/ml)**	135 (17–444)	84 (20–263)
**AST (U/ml)**	80 (25–251)	64 (24–255)
**γGT (U/ml)**	87 (16–347)	70 (9–205)

Characteristics of chronic HCV patients studied: HCV genotype, HCV-RNA load, serum IP-10 levels, the levels of liver enzymes ALT, AST and γGT. The average values and the range are presented.

### Determination of HCV load and liver enzymes

Quantification of HCV RNA levels was assessed using the Cobas Amplicor HCV monitor test (Roche Diagnostics, Branchburg) according to the instructions of the manufacturer. The levels of γGT, ALT and AST were determined in serum using the COBAS Integra 400 plus analysis system.

### IL-28B gene associated polymorphisms in chimpanzees

To determine the genotype of the rs8099917 and rs12979860, genomic DNA was isolated from either chimpanzee PBMC or whole blood [26] and the nucleotide sequence was determined by PCR amplification followed by direct sequencing. To assess the genotype of rs8099917, a previously described set of PCR primers was used [27], while a new set of primers was designed for the amplification of a fragment of 209 bp containing rs12979860 (IL-28-860-F2 5′-GGACAAGCGGCGCTTATCG-3′ and IL-28-860-R2 5′-GGCTCCAGGTCGGG GCG-3′). To amplify the fragments of interest, 1 µg of genomic DNA, 0.5 µM of each PCR primer, 0.2 mM dNTP each, 1.5 mM MgCl_2_ and 1 unit of Platinum *Taq* DNA polymerase (Invitrogen, Paisley, Scotland) were mixed in the appropriate PCR buffer in a total volume of 50 µl. The PCR conditions were: 1 cycle of denaturation at 94°C for 2 min, 30 cycles of denaturation at 94°C for 30 s, annealing at 61°C for 30 s and extension of 68°C for 30 s, followed by a final extension for 7 min at 68°C. PCR fragments were purified, using the GenJET™ Gel Extraction Kit (Fermentas, St. Leon-Rot, Germany) according to the manufacturer's protocol. The nucleotide sequence of the PCR fragments was determined using the ABI Prism BigDye Terminator v3.1 Cycle Sequencing Kit (Applied Biosystems, Foster City, USA). 0.2 µM of PCR primer, 1 µl of BigDye Terminator-mix and 5× sequencing dilution buffer in a total volume of 10 µl and sequenced directly on an ABI 3130XL genetic analyzer (Applied Biosystems, Foster City, USA). The data was analyzed using MacVector™ version 12.0.2 (MacVector, Inc Cambridge, UK). Sequences observed for rs12979860 were deposited in the EMBL database and received the accession numbers **HE599784** and **HE599785**.

The genotypes of the IL28B associated SNPs in blood of patients were determined using competitive allele-specific PCR (KASP; Kbioscience Hoddesdon, UK).

### Serum levels of IP-10, IL-29 and IL-28A/B

The concentrations of IP-10, IL-29 and IL-28A/B in serum were determined using commercially available kits. The levels of IP-10 in patient- and chimpanzee sera were measured using the Quantikine Kit (R&D Systems, Abingdon, UK; sensitivity of assay: 2 pg/ml). The concentration of IL-29 and IL-28A/B in chimpanzee sera was measured using VeriKine-DIY Human Interferon Lambda immunoassays specific for IL-29 or for the combination of IL-28A/B (both from PBL InterferonSource, NY, USA). The assays were performed according to the manufacturer's instructions, and the detection limits for both immunoassays were 62.5 pg/ml. IL-29 ELISA results were confirmed by the human IL-29 ELISA Ready-SET-Go! kit (e-Bioscience, San Diego, USA, detection limit for this assay was 8 pg/ml).

### Statistics

Statistical analysis was performed using Graphpad Prism 5.0 (GraphPad Software, Inc., La Jolla, USA). To test if a significant correlation exists between two quantitative variables, the correlation coefficient r^2^ is calculated as a measure of variation of 2 variables. In addition, to test whether this correlation was significant, a t-test was performed. A two tailed T-test was used to determine statistical difference of liver-enzymes between patients and chimpanzees.
